# Study of the method of spinal cord neuron culture in Sprague–Dawley rats

**DOI:** 10.1002/ibra.12085

**Published:** 2022-12-25

**Authors:** Yi‐Fei Sun, Quan‐Yuan Chang, Narima Eerqing, Chang‐Yan Hu

**Affiliations:** ^1^ National‐Local Joint Engineering Research Center of Translational Medicine of Anesthesiology, Institute of Neurological Disease, West China Hospital Sichuan University Chengdu China; ^2^ Center for Epigenetics and Induced Pluripotent Stem Cells, Kennedy Krieger Institute Johns Hopkins University Baltimore Maryland USA; ^3^ Department of Anesthesiology Southwest Medical University Luzhou China; ^4^ Department of Physics and Astronomy University of Exeter Exeter UK; ^5^ Animal Zoology Department Kunming Medical University Kunming China

**Keywords:** Ara‐C, differential apposition method, natural growth, purification, spinal cord neurons

## Abstract

This study aimed to explore the method of culture of spinal cord neurons (SPNs) in vitro and to provide prerequisites for studying the molecular mechanism and pharmacological mechanism of spinal cord injury and repair. The spinal cord tissues of neonatal Sprague–Dawley rats were taken and digested by trypsin, followed by cytarabine (Ara‐C) to inhibit the proliferation of heterogeneous cells, differential velocity adhesion, and natural growth in neuron‐specific medium. Then, the morphology of SPNs was observed. Ara‐C treatment inhibited the growth of heterogeneous cells and the growth of spinal neurons. Using the differential velocity adhesion method, it was found that the adhesion time of heterogeneous cells and SPNs was not significantly different, and it could not separate neurons and heterogeneous cells well. A large number of mixed cells gathered and floated, and died on the 18th day. Compared with the 20th day, the cell viability of the 18th day was better (*p* < 0.001). The natural growth and culture of SPNs in Neurobasal‐A medium can yield neurons of higher purity and SPNs from the 12th day to the 18th day can be selected for related in vitro cell experiments.

## Graphical abstract

In this study, the separation and purification methods of spinal cord neuron (SPN) culture, that is, the cytarabine (Ara‐C) inhibition method, the differential velocity adherent method, and natural growth medium for neurons, were compared. The neuron‐specific medium can be grown naturally and cultivated to obtain SPNs of higher purity. Let them grow naturally, the heterogeneous cells appear aggregated and floated on the 11th day, and individual neurons can be clearly seen. On the 18th day, a large number of heterogeneous cells aggregated and died. This was especially obvious on the 20th day. Compared with the SPNs on the 18th day, the cell viability on the 18th day was better.

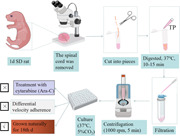

## 1 INTRODUCTION

The spinal cord is an extension of the central nervous system derived from the brain, a tubular neural structure located in the spinal canal, a lower center for many simple reflex activities, and its main function is to transmit neural information between the brain and the periphery. Spinal cord neurons (SPNs) are the most basic structural and functional units in the spinal cord, and are the terminal cells that carry out the instructions issued by the brain.^1^, ^2^ The pathological basis of anatomical spinal cord injury (SCI) is the death of SPNs and the loss of synaptic structure and function. Therefore, to better elucidate the molecular mechanism, pharmacological action mechanism, and drug effect identification of SCI and repair, in vitro culture of SPNs is essential,^3^ and primary cultures of SPNs can be very similar to in vivo.

Primary culture of SPN refers to the culture of cells or tissues obtained from living organisms in vitro.^4^ The experimental conditions are stable and controllable, and the experimental environment is relatively simple. In addition, observation and detection can eliminate the interference of many factors such as circulation, endocrine, and other cells, and have become an indispensable model tool in neuroscience research.^5^ SPN is a highly differentiated terminal cell that cannot divide and proliferate. It has high nutritional requirements and is extremely sensitive to environmental changes. In addition, the spinal cord contains a large number of glial cells, some fibroblasts, and a small number of neurons. It is difficult to isolate and purify. Therefore, the establishment of a stable and efficient method for in vitro culture of SPN is a prerequisite for SCI research. We explore the method of isolation, purification, and culture of SPN, optimize the culture system, and provide qualified cells for in vitro basic experiments related to SPN (such as SCI). At present, there are many purification methods in primary culture, such as the cytarabine (Ara‐C) inhibition method, the differential velocity adherent method, and natural growth of neurons in special medium, but few studies have compared their purification efficiency and quality.^6^, ^7^, ^8^

In this study, on the basis of the relevant literature, the primary culture and purification steps were compared with the Ara‐C inhibition method, the differential velocity adherent method, and natural growth medium for neurons to determine which separation and purification method was more effective in the culture of SPNs.

## 2 MATERIALS AND METHODS

### 2.1 Experimental animals

Neonatal Sprague–Dawley (SD) rats were provided by the Department of Laboratory Zoology, Kunming Medical University, and all experiments were approved by the Ethics Committee of Kunming Medical University (Approval No. kmmu20220795).

### 2.2 Reagents and instruments

Neurobasal‐A medium, B27, GlutaMAX‐L, trypsin (TP), and fetal bovine serum (FBS) were purchased from Gibco Company; Dulbecco's modified Eagle's medium (DMEM) (high glucose, containing sodium pyruvate) was purchased from Biosharp Company; Penicillin–Streptomycin (PS) was purchased from Hyclone Company; Poly‐l‐lysine (PLL) was purchased from Solarbio Company; Neuronal β‐III Tubulin (Tuj1) antibody was purchased from abcam Company; and antifluorescence Quenching mounting medium and 4’,6‐diamidino‐2‐phenylindole (DAPI) were purchased from Beyotime Company. B2 The Biosafety cabinet (Thermo), dissecting microscope, and inverted phase‐contrast microscope were manufactured by Nikon Corporation.

### 2.3 Configuration of culture medium

Basal medium: 89% DMEM + 10% FBS + 1% PS; Neuron‐specific medium: 97% Neurobasal‐A + 2% B27 + 1% GlutaMAX‐L + 0.5% PS.

### 2.4 Pretreatment of culture plates

The plates were wrapped with 1× PLL at 37°C for 2 h before sampling and then recovered, and they were air‐dried in a 37°C, 5% CO_2_ incubator for later use.

### 2.5 Isolation of SPNs

The neonatal 24 h SD mammary rats were disinfected with a 75% alcohol spray and soaked for 3~5 min and then decapitated and killed. The epidermal tissue was peeled off along the dorsal midline, the upper end of the spinal cord was held with forceps, the upper dorsal root of the spinal column was separated with scissors, care was taken not to cut the viscera, the whole spinal column was separated and placed into a Petri dish containing phosphate‐buffered saline (PBS), the spinal cord was blown out of the spinal cord cavity with a 10 ml syringe from the lumbosacral section of the spinal column, the spinal cord was immediately placed in a pre‐chilled DMEM high‐sugar medium, and the vascular membrane of the spinal cord was peeled off under a dissecting microscope. The stripped spinal cord tissue was placed in a 5 ml EP tube, cut into small pieces of 1 mm^3^ size with microscopic scissors, and digested by adding 1.5 ml of 0.25% TP at 37°C in a 5% CO_2_ incubator for 10–15 min, shaking once every 5 min. After digestion, 2 ml of basal medium was added to terminate the digestion. The cells were gently blown 30 times with a barrel and held for 2 min. The supernatant was obtained and filtered into a 50 ml centrifuge tube with a 40 μm cell strainer, and then 3 ml of DMEM was added to the precipitated tissue mass. The above blowing steps were repeated and the cell suspension was aspirated and filtered; this was again repeated three times, centrifugation was performed at 1000 r.p.m. for 5 min, and the supernatant was removed. The cells were resuspended by adding the basal medium and then counted; the cell density was adjusted to 1.3 × 10^6^/ml. The cell suspension was inoculated into a culture plate (100 μl/well for 96‐well plate, 500 μl/well for 24‐well plate, 2 ml/well for 6‐well plate) and incubated at 37°C and 5% CO_2_ in an incubator; the basal medium was changed to neuronal medium after 4 h of incubation. After that, the half medium was changed every 3 days (d).

### 2.6 Purification of SPNs

#### 2.6.1 Ara‐C treatment

As the SPNs cultured according to method 2.5 at 10 d had many heterogeneous cells and low neuronal purity, to improve the purity of neurons, we tried to use Ara‐C to inhibit the division and proliferation of heterogeneous cells. We first treated the cells with 3 μg/ml of Ara‐C for 48 h after 24 h of inoculation and then changed the solution. The concentration of Ara‐C was later adjusted to 1 μg/ml on the third and fifth day of cell inoculation. Ara‐C (1 μg/ml) was found to inhibit the growth of heterogeneous cells, but neurons died later, so we used different concentrations of Ara‐C at different time points of treatment for 48 h and then changed the solution, that is, we used 0.3, 0.6, 0.8, and 1 μg/ml of Ara‐C added at 4, 24, 48, and 72 h of cell inoculation, respectively.

#### 2.6.2 Differential velocity adherent

After treatment with Ara‐C, we found that the damage to neurons was also high, and then we used differential adhesion for culture. We extracted neuronal cells as in method 2.5, then inoculated the cells in PLL‐coated and uncoated 96‐well plates, and transferred the original supernatant into new PLL‐coated wells at 0.5, 1, 1.5, 2, 2.5, 3, 3.5, and 4 h after inoculation. After that, the half liquid was changed every 3 d.

#### 2.6.3 Natural growth

After Ara‐C treatment, the viability of neuronal cells was poor and heterogeneous cells still existed. Considering that we used a neuron‐specific medium during the culture process, it should not be suitable for the growth of heterogeneous cells. We continued to culture the cells and changed the medium every 3 d until 22 d.

### 2.7 Immunofluorescence identification

The cultured SPNs were washed once with PBS and fixed with 4% paraformaldehyde for 15 min, washed three times with PBS for 5 min/time, then permeabilized and blocked with a 0.3% Triton solution and 5% goat serum at 37°C for 30 min, followed by aspiration of the liquid and incubation with anti‐Tuj1 mouse monoclonal antibodies (ab78078, 1:250) at 4°C overnight. Subsequently, after washing three times with PBS for 5 min/time, the samples were incubated with secondary antibodies. Dylight 488 goat antimouse IgG (A23210, 1:250, Abbkine) was incubated at 37°C for 1 h, then washed with PBS three times (5 min/time), and DAPI was added to counterstain the nuclei. The fluorescent images were observed using a Nikon three‐dimensional inverted phase‐contrast microscope.

### 2.8 Cell Counting Kit‐8 (CCK‐8) assay

SPNs were inoculated in 96‐well culture plates (1.3 × 10^5^/well) and incubated in a 5% CO_2_ incubator at 37℃ for 18 and 20 d. Neurons were washed with PBS once, 100 μl of neuron medium and 10 μl of CCK‐8 solution were added into each well of the 96‐well plate to be tested, and incubated at 37℃ for 2 h. A microplate reader (BioTek Instruments, Inc.) was used to determine the OD values in each experimental well at 450 nm and to detect changes in cell proliferation capacity in each group.

### 2.9 Statistical analysis

All data in the experimental process are presented as mean ± SD and plotted using Graph Pad Prism 8 (Graph Pad Software). SPSS 21.0 was used for statistical analysis. Differences in cell viability between 18 and 20 d were analyzed using a *t* test of independent samples. The data were normally distributed. First, the Levene test was used to detect homogeneity of variance, and then the results of homogeneity of variance were used to determine whether the difference was statistically significant. *p*  <  0.05 indicated a significant difference.

## 3 RESULTS

### 3.1 In vitro culture of SPNs

**Figure 1:**
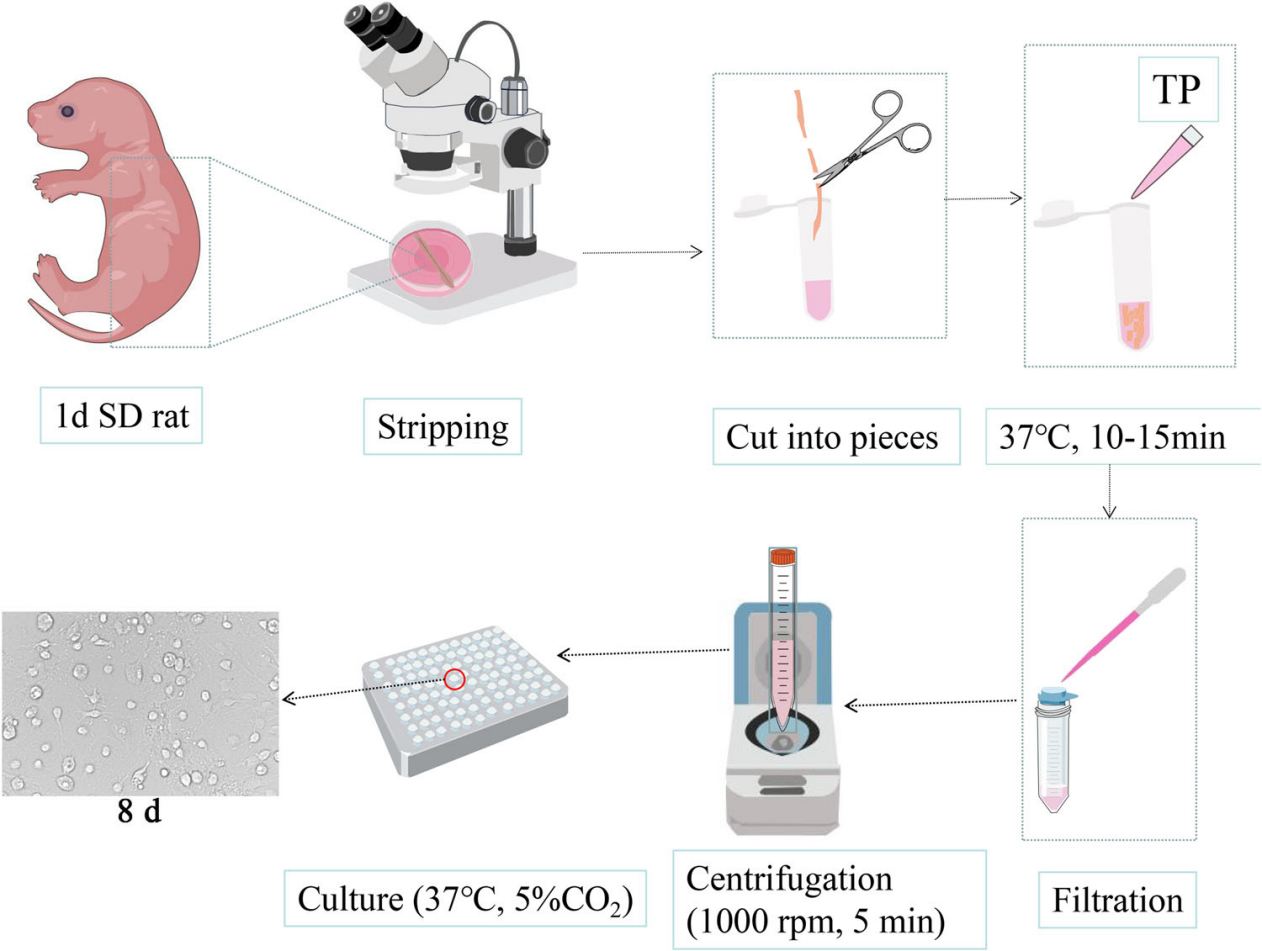
Flow chart of spinal cord neuron culture. d, day; min, minutes; SD, Sprague–Dawley; TP, trypsin. [Color figure can be viewed at wileyonlinelibrary.com]

The spinal cord was isolated by disinfection with 75% ethanol, decapitation, and killing of 1 d SD rats, digested with 0.25% TP for 10–15 min, centrifuged, and then inoculated using basal medium. Many stray cells and low purity of neurons were observed, as shown in the flow chart of SPN culture in Figure 1.

### 3.2 Purification of SPNs by Ara‐C treatment

**Figure 2:**
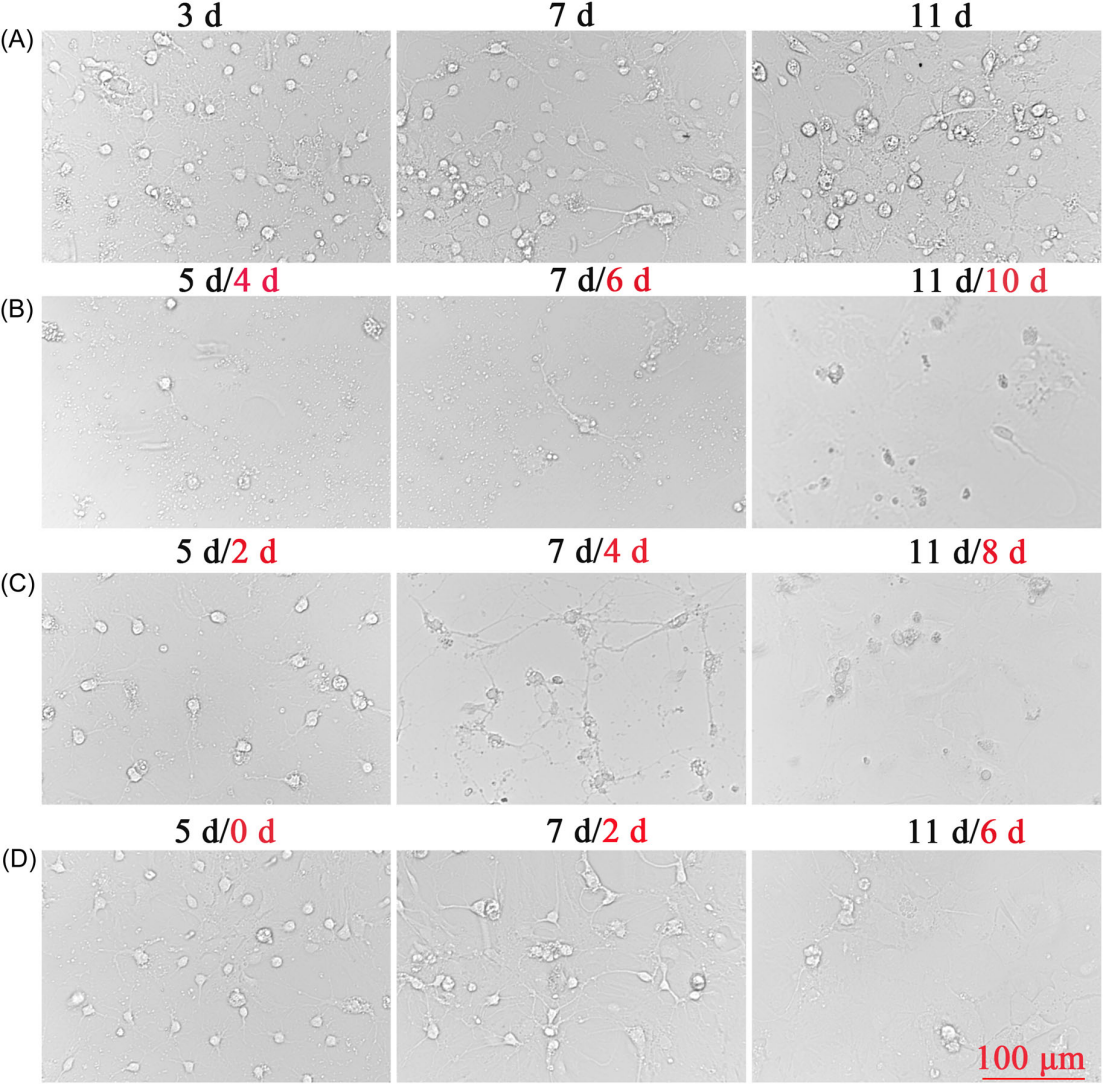
Spinal cord neurons (SPNs) treated with cytarabine (Ara‐C). (A) Untreated SPNs at 3, 7, and 11 d. (B) SPN cells treated with 3 μg/ml Ara‐C (1 d after inoculation) at 5 d (4 d), 7 d (6 d), and 11 d (10 d). (C) SPN cells treated with 1 μg/ml Ara‐C (3 d after inoculation) at 5 (2 d), 7 (4 d), and 11 d (8 d). (D) SPN cells treated with 1 μg/ml Ara‐C (5 d after inoculation) at 5 (0 d), 7 (2 d), and 11 d (6 d); the black font represents days of growth of SPNs and the red font represents SPNs after Ara‐C treatment. Scale bar = 100 μm. d, days. [Color figure can be viewed at wileyonlinelibrary.com]

**Figure 3:**
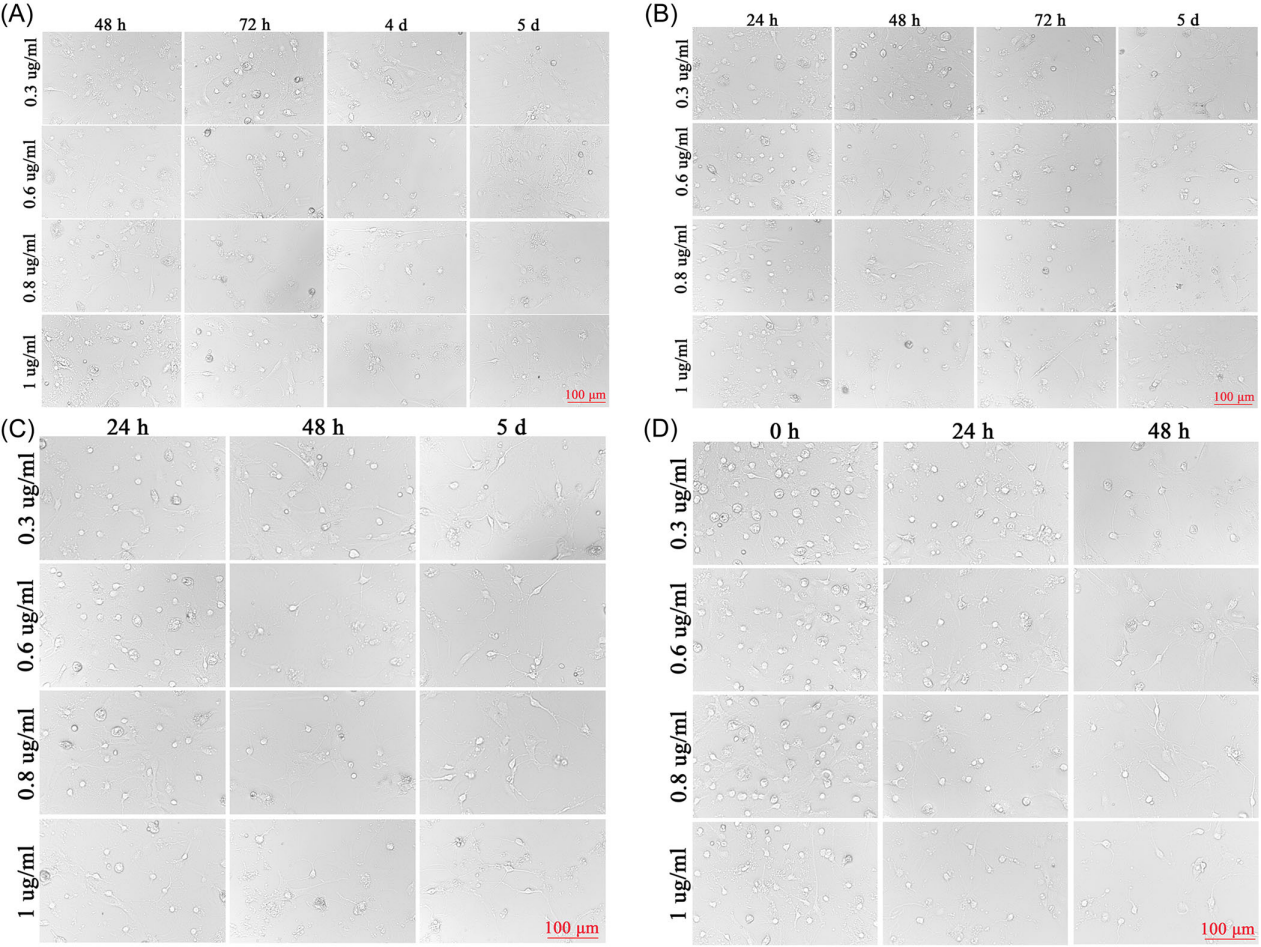
Changes in spinal cord neurons treated with different concentrations of Ara‐C at different times. (A) Neurons treated with Ara‐C (0.3, 0.6, 0.8, and 1 μg/ml) at 48 h, 72 h, 4 d, and 5 d. (B) Neurons treated with Ara‐C (0.3, 0.6, 0.8, and 1 μg/ml) at 24 h, 48 h, 72 h, and 5 d. (C) Neurons treated with Ara‐C (0.3, 0.6, 0.8, and 1 μg/ml) at 24 h, 48 h, and 5 d. (D) Neurons inoculated with Ara‐C (0.3, 0.6, 0.8, and 1 μg/ml) at 0, 24, and 48 h. Scale bar = 100 μm. d, days. [Color figure can be viewed at wileyonlinelibrary.com]

When the SPNs were first inoculated, the cells were round, translucent, and well refracted. Four hours after inoculation, most of the neuronal cells were already attached to the wall, the cells were round, small, and transparent, and synapses had not yet grown. After 1 d of inoculation, some synapses were visible, but the synapses were short and few. SPNs treated with not Ara‐C at 3, 7, and 11 d in Figure 2A had significantly more mottled cells compared with those treated with Ara‐C in Figure 2B–D, but the morphology and status of cells without Ara‐C treatment were better. Compared with 3 μg/ml Ara‐C treatment as shown in Figure 2B and 1 μg/ml Ara‐C treatment as shown in Figure 2C, D, a large number of cells died, and the structure of SPNs was basically incomplete and in a poor state. Two days after 1 μg/ml Ara‐C treatment (Figure 2C), it was observed that there were few miscellaneous cells but the number of neurons was less and the cell was in a poor state compared with Figure 2A, and the cell morphology continued to change and the number of cells continued to decrease after the fluid change. Before and after treatment with Ara‐C, the reduction of heterogeneous cells was not obvious, but the status of neuron was affected (Figure 2D). At 4, 24, 48, and 72 h after seeding, SPNs were treated with 0.3, 0.6, 0.8, and 1 μg/ml of Ara‐C, respectively. Ara‐C did not have a good inhibitory effect on hybrid cells and damaged neurons, as shown in Figure 3A–D.

### 3.3 Differential adherent culture of SPNs

**Figure 4:**
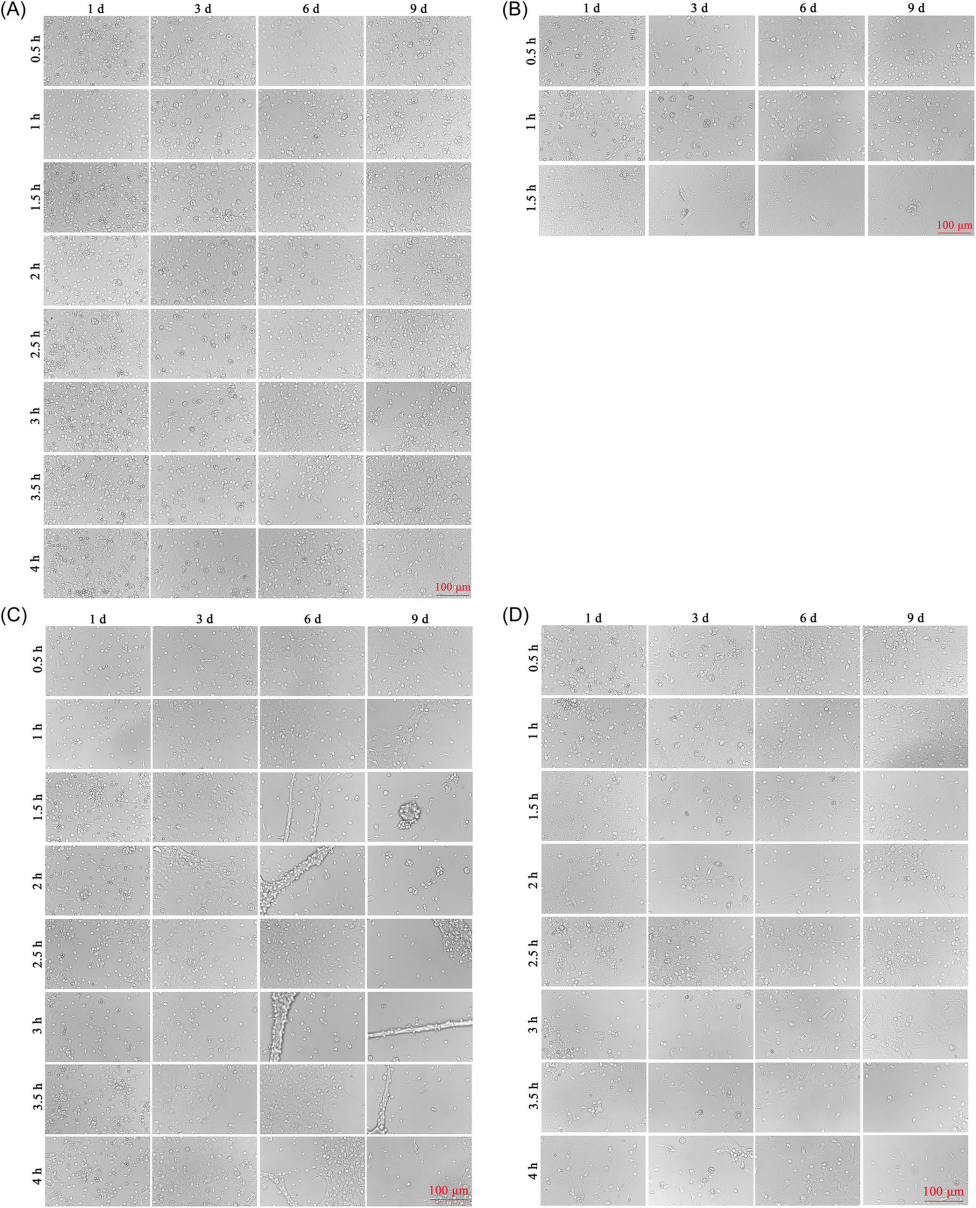
Morphology of spinal cord neurons after different time differential velocities. Cells remaining in plates with PLL coating after 0.5, 1, 1.5, 2, 2.5, 3, 3.5, and 4 h differential. (B) The cells of plates with PLL after 0.5, 1, and 1.5 h differential. (C) Original cells in culture plates not coated with PLL after 0.5, 1, 1.5, 2, 2.5, 3, 3.5, and 4 h differential. (D) Cells after differential velocity of 0.5, 1, 1.5, 2, 2.5, 3, 3.5, and 4 h without PLL were inoculated with PLL; 1, 3, 6, and 9 d was the time after the cell inoculation. Scale bar = 100 μm. d, days. [Color figure can be viewed at wileyonlinelibrary.com]

The differential adhesion method was used to culture neurons. It was found that almost all cells in PLL‐coated culture plates adhered to the wall after 1 h of cell inoculation, and there were almost no cells in the supernatant after 1.5 h. There was no significant difference between the neuronal heterocells at 0.5 and 1 h, and the liquid exchange at 4 h. After 0.5 to 4 h of differential velocity, there were cells in the culture plates without PLL coating, but the number of cells was less than that of the original culture plate, and there were still heterogenous cells (Figure 4).

### 3.4 Naturally growing SPNs

**Figure 5:**
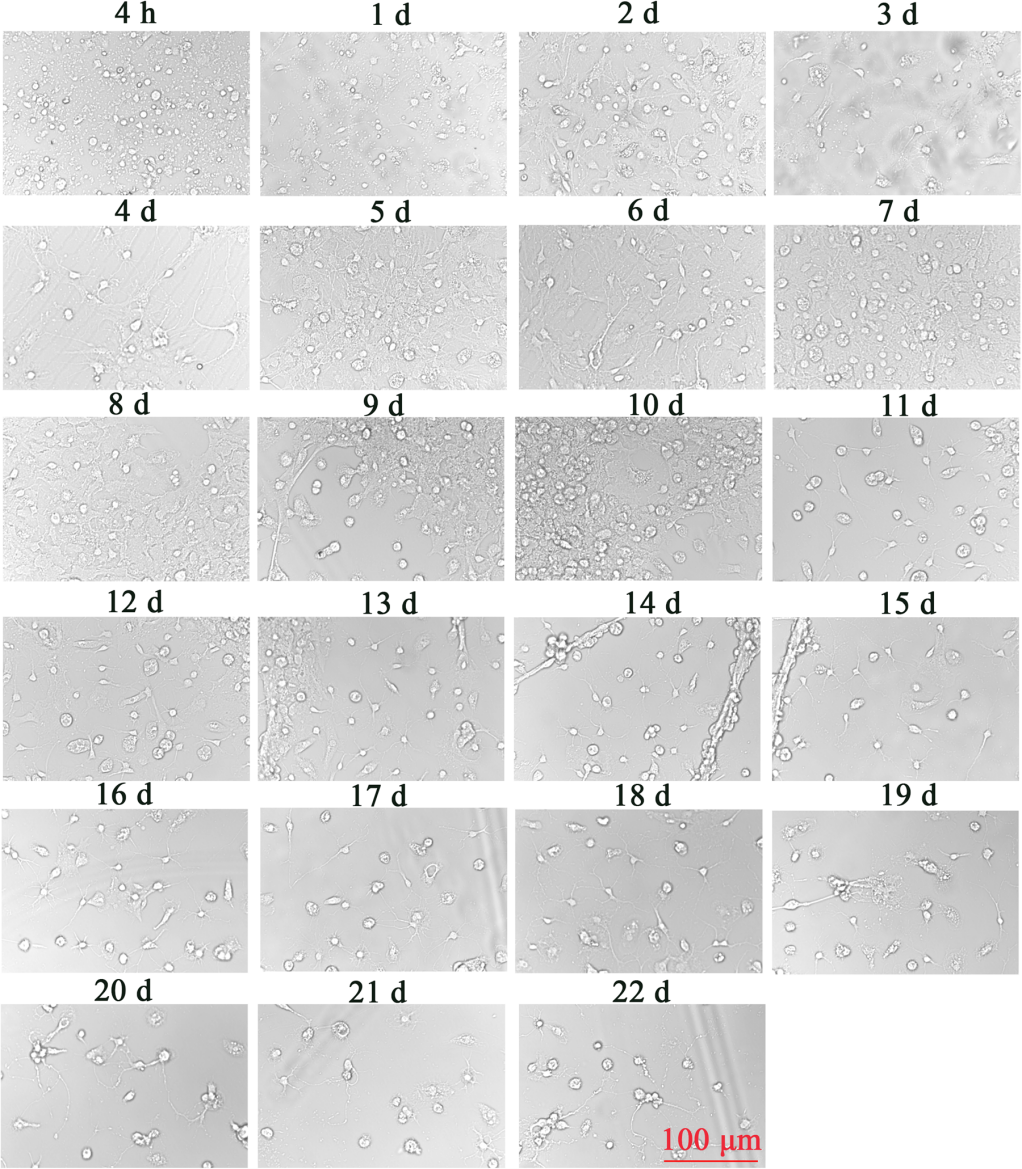
Spinal cord neurons at different times. Bright‐field morphology of spinal neurons of 4 h to 22 d. Scale bar = 100 μm. d, days. [Color figure can be viewed at wileyonlinelibrary.com]

**Figure 6:**
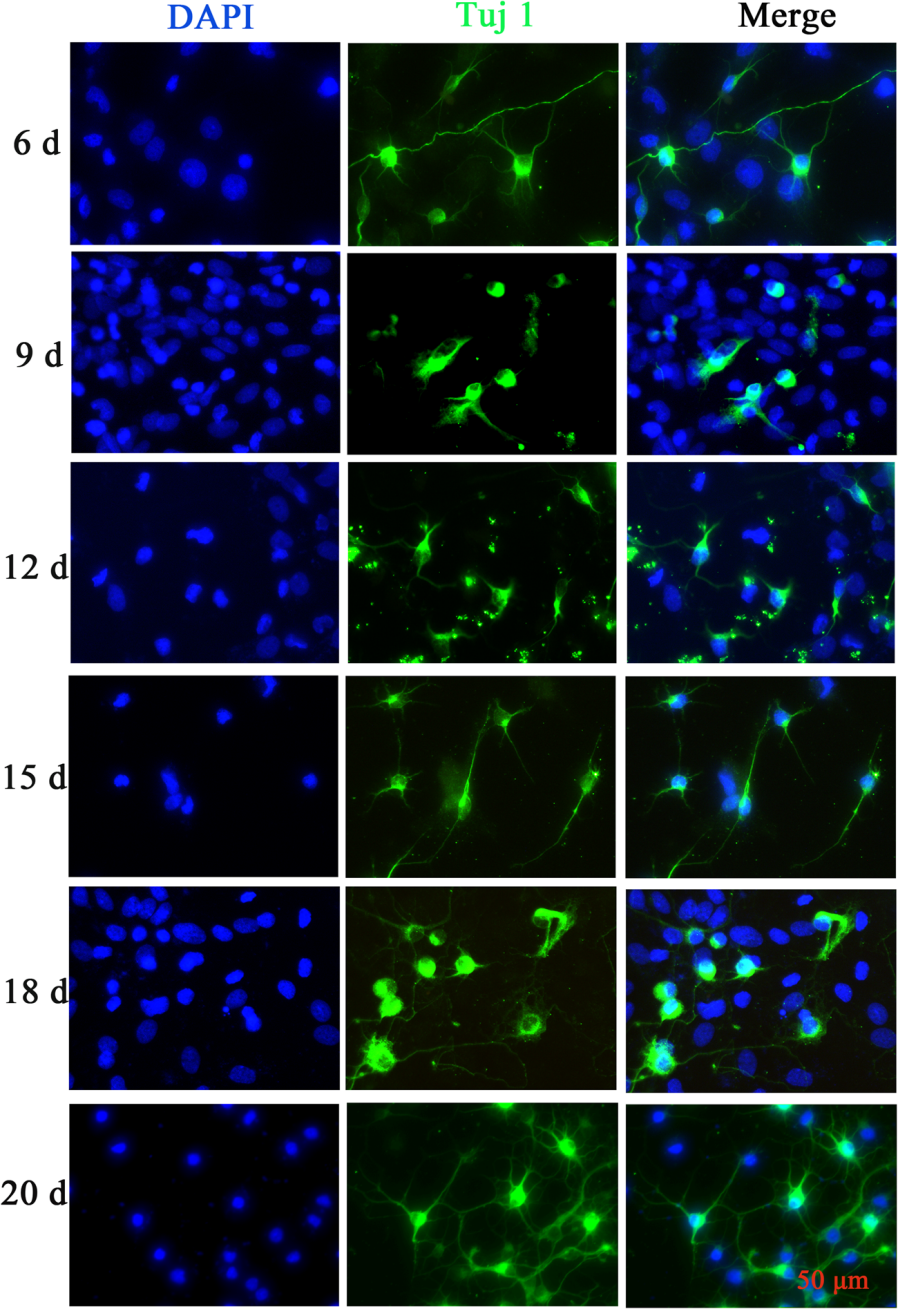
Immunofluorescence map of spinal cord neurons (SPNs). Immunostaining assay for the neuronal marker Tuj1 in SPNs at Day 6, 9, 12, 15, 18, and 20. Scale bar = 50 μm. d, days; DAPI, 4’,6‐diamidino‐2‐phenylindole; Tuj1, Neuronal β‐III Tubulin. [Color figure can be viewed at wileyonlinelibrary.com]

**Figure 7:**
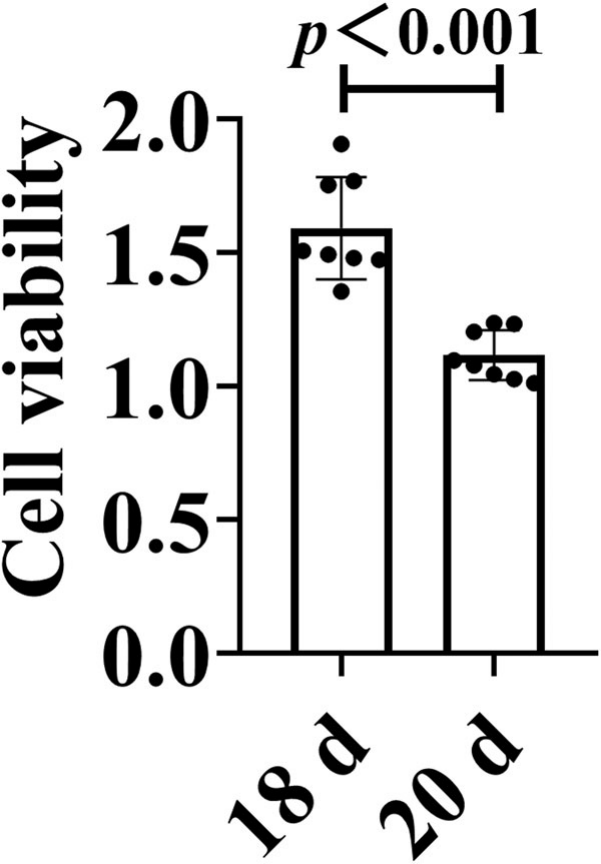
Cell Counting Kit‐8 (CCK‐8) viability test of spinal cord neurons. Spinal cord neurons at 18 days were more active than those at 20 d, *p* < 0.001; d, days.

On the 1st to 4th day after seeding of spinal neurons, there were few heterocytes; on the 5th to 10th day, a large number of heterocytes proliferated to cover neurons; on the 8th day, a small number of heterocyte cells were observed to aggregate; on the 11th day, the heterogeneous cells appeared aggregated and exposed and a single neuron was obviously visible; and on the 18th day, the axons of neurons aged and broke, and the cell state gradually deteriorated. After 22 d, the cells aged and axons disappeared (Figure 5). Immunofluorescence results showed a relative decrease in the number of parenchyma cells and an increase in the purity of naturally growing spinal neurons after 12 d (Figure 6). The cell viability of SPNs cultured for 18 d was better than that of SPNs cultured for 20 d (*p* < 0.001) (Figure 7).

## 4 DISCUSSION

In this experiment, we compared the culture methods of three kinds of SPNs, namely Ara‐C treatment, differential velocity adherent, and natural growth, and found that different concentrations of Ara‐C treatment at different cell seeding times did not significantly inhibit the growth of heterocells. The purification rate of neurons was not high, and the neurons showed toxicity to cells, inhibiting their growth and activity and damaging the cell structure. Using the differential velocity adherent method, the adhesion rates of SPNs and heterocysts were not much different, and they all adhered to the wall in almost the same time period, so they could not be separated, and the purification effect was not ideal. The neuron‐specific medium was used to allow it to grow naturally. On the 5th–10th day, the heterogeneous cells proliferated and covered SPNs, and then agglomerated. On the 12th–18th day, a large number of heterogeneous cells were removed from the supernatant to achieve the purification effect. At the same time, neurons could grow naturally, with better activity and complete axons before the 18th day.

### 4.1 Spinal cord tissue acquisition

In the rat spinal cord, there are various types of cells: neurons, oligodendrocytes, astrocytes, microglia, fibroblasts, and endothelial cells.^9^ The primary culture of SPNs will inevitably be contaminated by other cell types, so in our method of isolating and purifying SPNs from the spinal cord, we use a dissecting microscope to carefully dissect the tiny capillaries and dura from the spinal cord to reduce contaminating cells, because fibroblasts and endothelial cells are the two major cellular components of the meningeal covering and accompanying tiny capillaries.^10^ When we stripped the tiny capillaries and dura as much as possible we can effectively reduce or even remove fibroblasts and endothelial cells from the culture. In addition, to sufficiently dissociate cells and harvest more viable SPNs, we chose to trypsinize tissue. While trypsinization is acute, it can cause direct damage and irritation to dissociated tissue, so we use a low concentration of TP, which leads to relatively mild digestion and fewer dead cells. By controlling the pH, temperature, concentration, and time in real time, overdigestion and cell damage can be avoided.^11^, ^12^ Papain has also been widely used to digest and culture primary neurons.^13^, ^14^ Studies have compared the digestion efficiency and culture effect of TP and papain, and found that it is comparable to that of papain. Compared with the papain group, the TP group had more cortical neurons, larger cell bodies, longer axonal lengths, and fewer impurities.^15^

### 4.2 Ara‐C kills dividing and proliferating cells

Ara‐C is an antimetabolite that affects pyrimidine metabolism, which is catalyzed by deoxycytidine kinase to arabinosine diphosphate or glycytidine triphosphate in vivo, which can inhibit the activity of DNA polymerase and interfere with nucleotide incorporation into DNA. Inhibition of nucleotide reductase and blocking of the reduction of cytosine nucleotide to deoxycytosine nucleotide, which specifically acts on the S phase, thus affect DNA synthesis in the S phase of dividing cells and kill dividing cells.^16^, ^17^ Neuronal cells are highly differentiated cells that do not proliferate, and Ara‐C has almost no direct inhibitory effect on their growth, but can disrupt the neuronal cell growth environment by inhibiting the migration and proliferation of non‐neuronal cells, leading to a decrease in the secretion of trophic factors such as the glial cell line‐derived neurotrophic factor (GDNF) in non‐neuronal cells, which disrupts the neuronal cell growth environment and thus indirectly affects the growth of SPN cells.^18^ Therefore, the rational use of Ara‐C is the key to purification. If Ara‐C is added too early or at too high a concentration, the coexistence environment between neurons and glial cells will be destroyed, glial cells will die in large numbers, and the GDNF secreted by glial cells to promote neuronal survival and morphological differentiation will be markedly reduced; if it is added too late or at too low a concentration, glial cells will divide and proliferate in large numbers and consume a large amount of nutrients in the culture medium, which will also inhibit the survival of spinal neurons. The continuous presence of Ara‐C in the culture system also exerts toxic effects on neurons.^19^, ^20^ We tried for the first time to treat SPNs at 4, 24, 48, and 72 h of inoculation with concentrations of 0.3, 0.6, 0.8, and 1 μg/ml of Ara‐C, respectively, but low concentrations of Ara‐C did not inhibit promiscuous cells well and may also damage neurons by prematurely disrupting the normal physiological environment of neurons. Therefore, Ara‐C is not a good choice in the purification process of SPNs.

### 4.3 Differential velocity adherent with the PLL coating

The differential velocity adherent method is a purification method that induces little damage. It achieves the purpose of separating and purifying neurons according to the different adhesion ability of various cells in nerve tissue.^21^ In the culture system, the most easily adherent cells are fibroblasts, followed by glial cells, and finally neurons.^22^ PLL encapsulation can improve the adhesion ability of cells.^23^, ^24^ Therefore, we assume that PLL has different adhesion ability to different cells and will separate fibroblasts and other heterocytic cells. However, it was observed during the experiment that PLL‐coated pore cells were basically stuck to the wall after 0.5 h of inoculation, and only a small number of cells and few neurons were observed after the replacement supernatant was re‐inoculated. Therefore, this method is not very useful for purifying neurons.

### 4.4 Natural growth

Neurobasal‐A without serum medium is dedicated to neuronal culture with B27 nerve growth factor. It prevents the growth of glial cells and other non‐neuronal cells, promotes neuronal growth, and prolong neuronal survival time.^25^ In the process of culture, we changed the amount of fluid by half every 3 days to remove the suspended cells with inhibited growth, leaving purer neurons and improving the purity of neurons.

In conclusion, SPNs naturally grew in Neurobasal‐A medium with B27 to obtain high purity neurons, without adding Ara‐C and differential adherent operation, which is easy to operate. SPNs on Days 12–18 can be selected as experimental cells.

## AUTHOR CONTRIBUTIONS

Chang‐Yan Hu and Narima Eerqing conceptualized the idea of the study. Yi‐Fei Sun and Quan‐Yuan Chang completed the experiment, analyzed the data, performed document retrieval, and revised and polished the manuscript.

## CONFLICT OF INTEREST

The authors declare no conflict of interest.

## ETHICS STATEMENT

This study was approved by the Ethics Committee of Kunming Medical University (Approval No. kmmu20220795).

## Data Availability

The data of our study are available on reasonable request.
